# Role of Regenerating Islet-Derived Protein 3A in Gastrointestinal Cancer

**DOI:** 10.3389/fonc.2019.01449

**Published:** 2019-12-17

**Authors:** Meng-ya Zhang, Jun Wang, Jie Guo

**Affiliations:** ^1^Hubei Province Key Laboratory of Occupational Hazard Identification and Control, Wuhan University of Science and Technology, Wuhan, China; ^2^Department of Pharmacy, New Medicine Innovation and Development Institute, College of Medicine, Wuhan University of Science and Technology, Wuhan, China

**Keywords:** regenerating islet-derived protein 3A, gastrointestinal cancer, proliferation, apoptosis, inflammation, invasion

## Abstract

Regenerating islet-derived protein 3A (Reg3A), a protein mainly expressed in the digestive system, has been found over-expressed in many kinds of gastrointestinal cancer, including hepatocellular carcinoma, pancreatic cancer, gastric cancer, and colorectal cancer, therefore has been considered as a promising tumor marker. In recent years, considerable attention has been focused on the tumorigenesis effects of Reg3A, which were mainly manifested as cell proliferation promotion, cell apoptosis inhibition, the regulation of cancer cell migration and invasion. In particular, based on the significant up-regulation of Reg3A during pancreatic inflammation as well as its tumorigenic potential, Reg3A has been considered to play a key role in inflammation-linked pancreatic carcinogenesis. In addition, we here systematically generalized the reported Reg3A-related signaling molecules, which included JAK2-STAT3- NF-κB, SOCS3, EXTL3-PI3K-Akt, GSK3β, Wnt/β-catenin as well as some invasion and migration-related genes (Snail, MMP-2, MMP-9, E-cadherin, RhoC, and MTA1). And gp130, EGFR, EXTL3, and Fibronectin 1 might act as potential receptors for Reg3A.

## Introduction

Regenerating islet-derived protein 3A (Reg3A), a 19 kD secreted calcium-dependent lectin protein, belongs to the Reg (Regenerating) family which includes four subclass (Reg1A, Reg1B, Reg3A, and Reg4) in humans based on the primary structures of DNA sequence and protein (1, 2). Owing to the nomenclatural redundancy of Reg proteins, Reg3A is also known as Reg3α or Reg III (2, 3). And the murine homolog of human Reg3A gene is Reg3γ or Reg3g (4). It is generally considered that Reg3A protein is selectively expressed mainly in gastrointestinal organs, including the pancreas, the small and large intestinal tracts, etc. at relatively low levels under normal condition. Thereinto, the pancreas has the highest levels of Reg3A structural gene expression. For example, the level of Reg3A gene in mouse pancreas is about 19-fold higher than that in any other tissues such as duodenum (5). Within the pancreas, a more broad expression of Reg3A has been found in the acinar cells as well as the islet a-cells (5). Nevertheless, several studies (6, 7) indicated that Reg3A was undetectable in normal human pancreas.

Under physiological conditions, the antimicrobial role of Reg3A in the digestive system has been shown to be crucial for the regulation of host-microbiota interplay. Vaishnava et al. (8) reported that epithelial Reg3A was indispensable for maintaining a ~50-μm zone that physically contributed to the spatial segregation of epithelial surface and intestinal microbiota. In the absence of Reg3A, loss of host-bacterial segregation was coupled to increased bacterial colonization, enhanced microbiota-induced activation of intestinal adaptive immune responses as well as aggravated mucosal inflammatory (8, 9). Whereas, Reg3A overexpression restricted the bacterial colonization of mucosal surfaces, and protected experimental animals from dysbiosis-related digestive system disorders such as colitis (10) and alcoholic steatohepatitis (11).

Possible acting as a compensatory factor, Reg3A could be significantly up-regulated during digestive system infection, inflammation, and other disease conditions. As indicated by its name, Reg3A was first identified by its strong induction in regenerating pancreatic islets in response to stress/damage (1). Moreover, this secreted C-type lectin protein was found to be largely released by the pancreatic acini during acute and chronic pancreatitis, thus also named as pancreatitis-associated protein (PAP) (2). The involvement of Reg3A has been demonstrated in several disease conditions, such as pancreatitis (3), diabetes (12), skin inflammation and injury (12–14), and inflammatory bowel disease (11, 15). The potential protective activity of Reg3A against damage, as well as its antimicrobial and anti-inflammatory properties have been identified in these non-malignant diseases, which have been reviewed in a recent-published work (16). However, it is worth noting that most of the interest has been focused on the close relationship between Reg3A and gastrointestinal cancer. Up to date, Reg3A has been considered as a promising tumor marker and a novel intervention target for many kinds of gastrointestinal cancer, including pancreatic, liver, gastric, and colorectal cancer.

## up-Regulation of Reg3A in Gastrointestinal Cancer

The earliest identified Reg3A-overexpressed gastrointestinal malignancy is liver cancer, so that Reg3A is also named as the encoded protein of genes expressed in heptocarcinoma-intestine-pancreas (HIP) (2). Although the expression of Reg3A was undetectable in normal and non-tumoral liver tissue, Reg3A mRNA was found expressed at a high level in the tumors of 7 of 29 (about 25%) human primary liver cancers (17). Cavard et al. (18) also reported that the mRNA and protein expressions of Reg3A were strongly induced both in human hepatocellular carcinomas and in hepatoblastomas. In a study of 265 surgically resected unifocal primary hepatocellular carcinomas (19), Reg3A was found to express in 97 (36.6%) samples, but not in any of 219 non-tumorous liver tissues.

However, the pancreatic-specific expression of Reg3A (5) let many researchers focus their attention on its relationship to the pancreatic cancer. Reg3A was found over-expressed in 79% (30/38) of pancreatic tissues from Japanese individuals with pancreatic ductal adenocarcinoma (7). And Reg3A over-expression in pancreatic cancer was significantly correlated with nodal involvement, distant metastasis and short survival (7). In the pancreatic juice of patients with pancreatic adenocarcinoma, the levels of Reg3A had been found to be elevated, approximately 24 times higher than in patients with other pancreatic diseases, 16 times higher than those with chronic pancreatitis (20). Patients with Reg3A levels ≥20 μg/ml in pancreatic juice were 21.9 times more likely to have pancreatic adenocarcinoma than those with levels <20 μg/ml (20). Fukushima et al. (21) also reported that the gene expression of Reg3A in parenchyma of human pancreatic cancer increased 130.6-fold compared to that of normal pancreas, and this increase was the most obvious among a total of 87 over-expressed genes in pancreatic cancer. In order to search biomarkers for pancreatic cancer that can facilitate to monitor treatment efficacy or early detection, Porterfield et al. (22) performed an in-depth LC-MS/MS analysis of the proteome in pancreatic ductal fluid from normal and pancreatic adenocarcinoma patients, and Reg3A was found increased in cancer ductal fluid compared to normal. The mRNA expression levels of Reg3A in tumor tissues from 36 Chinese patients with pancreatic cancer (23) were significantly higher in the patients with inflammation history than those without inflammation history, in tumors >3 cm than those ≤3 cm, in low differentiated tumors than high and middle differentiated tumors, and those in TNM stage III-IV than I-II. A recent study (6) analyzed the level of Reg3A in serum or plasma from 85 healthy donors or 166 patients with pancreatic ductal adenocarcinoma from three independent cohorts. Patients with high circulating Reg3A levels had overall shorter survival as well as poor surgical outcomes with reduced disease-free survival (i.e., the time before disease recurrence). These data suggested that, as a secreted molecule highly expressed in pancreatic cancer and related to the clinical outcome of patients, Reg3A might be an effective biomarker enabling the earlier diagnosis, earlier therapeutic intervention, prognosis and stratification of patients with this deadly disease.

The Reg3A expression had also been investigated in gastric cancer samples. Reg3A gene was clarified overabundant in the peripheral blood circulation from gastric cancer patients, but not expressed in peripheral blood mononuclear cells from healthy volunteers, thus might be a potential molecular marker for detection of gastric cancer cells in the peripheral blood circulation (24). Within the gastric mucosa in a *H. pylori*-infected and high-salt diet (two important risk factors for stomach carcinogenesis)-treated mouse gastric tumor model, Reg3g was found to be one of two candidate up-regulated genes (the other was Cd177) with the fold change of 6.1 (25). Auto-antibodies against Reg3A had been detected in none of the healthy donors but in 22.9% of the gastric cancer patients (26). Chen et al. (27) found that the mRNA levels of Reg3A were significantly elevated in gastric cancer tissues from 41 Chinese patients compared with the matched normal tissues, and the data originated from the gastric cancer cell line SGC-7901 also verified this result. Nevertheless, there appears to be a mixed expression profile of Reg3A in gastric cancer. An earlier study conducted by Choi et al. (28) showed that, the expression of Reg3A in human stomach mucosa was down-regulated to a significant extent in the most of primary human gastric carcinomas (20 out of 30; 67%). The reasons for the discrepancy had been explained by the possibility that the different stages of gastric cancer samples were used in different studies (27). In addition, the down-regulation of Reg3A in gastric cancer reported by Choi et al. (28) was considered to be associated with hypermethylation. The epigenetic inactivation of Reg3A was presumed to occur during tumorigenesis in gastric cancer. Therefore, further researches enrolling large clinical samples of gastric cancer patients and systemic epigenetic studies would help to clarify the expression status of Reg3A in gastric cancer.

Colorectal cancer is one of the most common gastrointestinal malignancies in the world, which caused ~500,000 deaths per year, and has been ranked third in terms of cancer death (29). Nagaraj et al. (30) had identified secreted Reg3A protein as a potential biomarker for the early detection of colorectal cancer. In comparing 79 colorectal tumors to their matched normal mucosas, Reg3A gene in colorectal cancer tissues was up-regulated by 23-fold (31). Ye et al. (32) reported that Reg3A expression at mRNA level was up-regulated in 70.7% (58/82) of the tested colorectal cancer specimens. Moreover, Reg3A expression level was markedly correlated with the tumor size, differentiation or tumor stage. In particular, it had been well-accepted that specific bacterial species played at least some roles in the initiation and/or progression of colorectal cancer. And up-regulation of Reg3A was found in the tumors with high-level colonization by *Fusobacterium*, a kind of colorectal cancer-associated bacteria (33).

Together, the significant up-regulation of Reg3A in gastrointestinal cancer implied that Reg3A could at least act as a biological marker for the clinical diagnosis of diseases.

## Potential Functions of REG3A During Tumorgenesis and Development of Gastrointestinal Cancer

Notably, Reg3A might be not only useful as a promising tumor marker, but also play a potential key role in the initiation and progression of gastrointestinal malignancy.

### Proliferation-Promoting Effect of Reg3A

Reg3A has long been believed as a potent proliferation promotor in non-tumor cells, including epidermal keratinocytes (14, 34), insulin-positive cells in pancreatic tissues (35) and hepatocytes (36). Therefore, based on its intrinsic proliferation-promoting activity, Reg3A over-expressed in gastrointestinal cancer could be speculated to allow uncontrolled tumor growth.

Accumulating evidence has clarified the role of Reg3A in the development of pancreatic cancer. Treating primary mouse acinar cells with 100 nM Reg3A promoted acinar-to-ductal metaplasia formation with concurrent activation of mitogen-acitvated protein kinase (MAPK), a well-accepted master regulator of cell cycle and proliferation (37). In an *in-vivo* study (4), the caerulein-induced chronic pancreatitis mouse model was co-injected for 16 weeks with dimethylbenzanthracene and p*Reg3g*, the latter of which was a lentivirus system encoding for murine Reg3g. No visually or histologically detectable tumors was found in the mice receiving dimethylbenzanthracene alone, however, a combination of dimethylbenzanthracene and 10^8^T p*Reg3g* induced visually recognizable tumors in the pancreas. Similarly, Reg3A *in vivo* promoted the formation of KRAS-induced early pancreatic intraepithelial neoplasia lesions, which were the histopathological hallmarks of the initiation of pancreatic carcinogenesis (38). Moreover, this study (38) using BrdU as a proliferative marker confirmed that Reg3A directly promoted the growth of pancreatic cells *in vitro* through inducing cell proliferation. In human pancreatic cancer cell lines, incubation with exogenous Reg3A dramatically promoted the cell proliferation, the soft-agarose colony forming ability, the transcript levels of cell cycle regulatory switch Cyclin D1, decreased cell numbers at G0/G1 phase, and increased cell numbers at S phase (23). Whereas, a decrease in proliferation was observed in Reg3A siRNA-treated pancreatic cancer cells (39).

Proliferation-promoting effect of Reg3A has also been confirmed to be involved in the development of other kinds of gastrointestinal cancer. Chen et al. (27) demonstrated the proliferation ability of gastric cancer SGC7901 cells was repressed following silencing of Reg3A. Transfection with siRNAs targeting Reg3A resulted in the inhibited proliferation of colorectal cancer LOVO and RKO cells (32). On the contrary, the proliferation abilities of colorectal cancer HT-29 and SW116 cells were enhanced by Reg3A overexpression. This study (32) also explore the effect of Reg3A silence in colorectal cancer cells on tumor growth in nude mice. At 46 days after the injection of LOVO or RKO cells stably transducted with Reg3A short hairpin RNA lentivirus, the weight and volume of Reg3A-silenced tumors were significantly smaller and lighter than those of control lentivirus-treated tumors. These data suggested that inhibition of Reg3A in colorectal cancer cells could repress cell proliferation *in vitro* and *in vivo* (32).

However, a contrary evidence was observed by a recent study (40), in which the transduction of lentivirus carrying the Reg3A gene into gastric cancer MGC-803 or BGC-823 cells was found to cause a significant decrease in call viability, indicating Reg3A overexpression suppressed the proliferation of gastric cancer cells. This finding appeared to be in contradiction with that from gastric cancer SGC7901 cells (27), which might be due to the difference in cell lines used. Therefore, further studies in more cancer cell lines are required to elucidate the exact effect of Reg3A on proliferation of gastrointestinal cancer cells.

### Anti-apoptotic Effect of Reg3A

Yin et al. (4) had demonstrated that 16-week administration of high dose p*Reg3g* in mice decreased the expression levels of caspase-3, a key enzyme in apoptosis execution, in the pancreas. In pancreatic cancer cell line SW1990 or BxPC-3, flow cytometry analysis showed a dramatically high level of apoptosis after silencing endogenous Reg3A using siRNA (39). The anti-apoptotic Bcl2 in SW1990 and BxPC-3 cells induced by exogenous Reg3A incubation was markedly decreased by knockdown of endogenous Reg3A (39). Similarly, Loncle et al. (38) activated the apoptotic program of pancreatic cancer MiaPaCa2 and Panc1 cells by serum starvation. At the same time, cells were incubated with or without the recombinant protein of Reg3A. The results showed that the Reg3A treatment for 48 and 72 h increased the resistance of both MiaPaCa2 and Panc1 cells to apoptosis as evidenced by the increased cell viability and the decreased caspase-3/7 activity. Besides, knockdown of Reg3A with siRNA in two colorectal carcinoma cell lines (LOVO and RKO) markedly increased the cell apoptotic ratio measured by Annexin V-PI staining (32). These findings suggested that the potential carcinogenic effect of Reg3A might be associated with its influence on cell apoptosis, and Reg3A might serve as an oncogene by protecting cancer cells from cell apoptosis.

### Regulation of Cancer Cell Migration and Invasion by Reg3A

The positive correlation between the expression level of Reg3A and the motility of digestive tumor cells has been identified. Wang et al. (41) explored the role of Reg3A in migration and invasion of hepatocellular carcinoma, and found that the positive expressions of Reg3A were significantly correlated with the vascular invasion of hepatocellular carcinoma tissues from 75 patients. Further siRNA-mediated loss-of-function experiments (41) showed that silencing Reg3A expression could inhibit the invasion and migration of hepatocellular carcinoma, which were detected using wound healing assay and 24-well transwell assay. The same analysis methods were applied in gastric cancer line SGC-7901 cells (27), and the results also revealed that Reg3A promoted the gastric cancer cell invasion and migration. Nigri et al. (6) used mouse PKA4 and human pancreatic PANC-1 cell lines cultured with media containing different concentrations of Reg3A recombinant proteins, and found that the migration and invasion of pancreatic cancer cells appeared to be enhanced by Reg3A in a dose-dependent manner. The lives and survival rates of patients with pancreatic ductal adenocarcinoma had been considered to be drastically influenced by neural remodeling and perineural invasion (PNI) (6). In view of this fact, *ex vivo* PNI assay was also included in this study (6) to explore the presence of Pk4A cells which had invaded and migrated within the nerve fibers. The results confirmed the enhancing effect of Reg3A on PNI and tumor aggressiveness.

### Reg3A and Inflammation-Linked Pancreatic Carcinogenesis

Known as pancreatitis-associated protein, Reg3A is sensitively and markedly induced after the onset of pancreatitis, which is manifested as the 200- to 300-fold increase in Reg3A expression level after even mild pancreatic inflammation (3). Based on the significant up-regulation of Reg3A during pancreatic inflammation as well as its tumorigenic potential, it is rational to speculate that Reg3A plays a role in inflammation-linked pancreatic carcinogenesis. Using the immortalized but not transformed pancreatic epithelial HPDE6c7 cell line with phenotype as normal pancreas cells, Wang et al. (23) verified the proliferation-promoting effect of exogenous Reg3A on normal pancreas cells, which was manifested as the increases in cell viability, S phase cell population and cyclin D1 expression in Reg3A-treated HPDE6c7 cells compared with the control, suggesting Reg3A-mediated malignant transformation of pancreatic epithelial cells. Furthermore, the mRNA and protein expression levels of endogenous Reg3A in HPDE6c7 cells were shown to be enhanced by the stimulation of interleukin-6, a well-known cytokine playing a key role in tumor-promoting effect of inflammation (39). In order to obtain direct evidence for the key role of Reg3A in inflammation-induced pancreatic cancer, Yin et al. (4) injected with p*Reg3g* accompanied by a mutagenic carcinogen dimethylbenzanthracene to C57BL/6 mice with chronic pancreatitis induced by caerulein. And the addition of high dose of p*Reg3g* were reported to develop recognizable tumors in pancreas in mice with chronic pancreatitis, which indicated that Reg3g expression exacerbated pancreatic cancer in inflammation-associated cancer progression. Therefore, the up-regulation of Reg3A might act as one of the engines for the transformation from pancreatitis to pancreatic cancer.

## REG3A-Related Molecules

**Figure 1:**
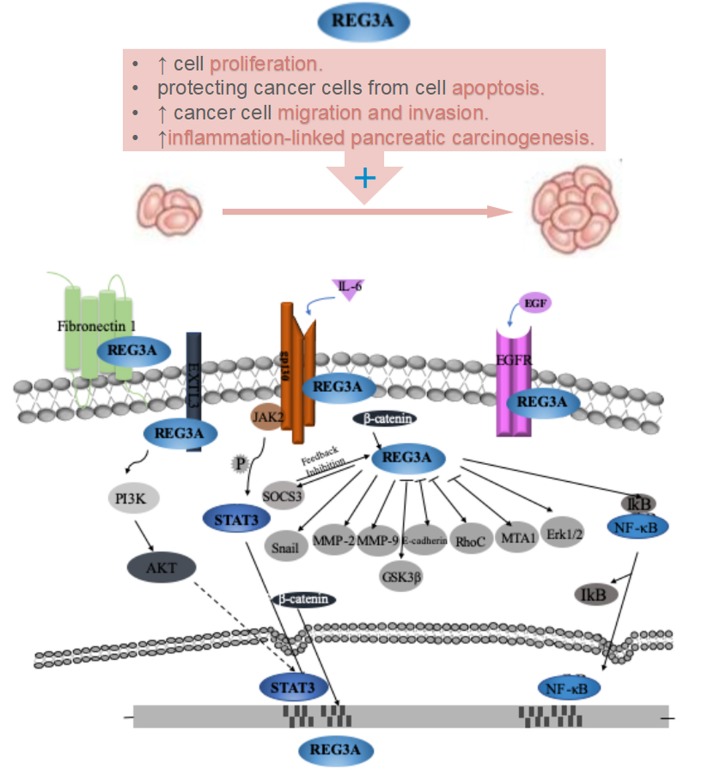
Role and its underlying mechanisms of Reg3A for carcinogenesis of gastrointestinal cancer.

In order to further clarify the role and molecular mechanisms of Reg3A in oncogenesis, the researches on Reg3A signal pathways have progressed in recent years, and many Reg3A-related molecules involved in initiation and progression of gastrointestinal cancer have been identified. As shown in Figure 1 and Table 1, more than 20 molecules have been reported to be involved in the Reg3A-related signal, which include JAK2-STAT3-NF-κB, SOCS3, EXTL3-PI3K-Akt, GSK3β, Wnt/β-catenin as well as some invasion and migration-related genes (Snail, MMP-2, MMP-9, E-cadherin, RhoC and MTA1). And gp130, EGFR, EXTL3, and Fibronectin 1 appeared to act as potential receptors for Reg3A. Especially, some uncovered molecules associating with cell proliferation and death (e.g., JAK2-STAT3-NF-κB pathway and its regulators gp130, SOCS3, EGFR) have been focused on and demonstrated to contribute to the mechanisms underlying the significant proliferation-promoting effect of Reg3A.

**Table 1:** Summary of Reg3A-related signal molecules.

**Potential role in Reg3A-related signal**	**Molecules**	**Main functions**	**Key evidences for correlation with Reg3A**	**Gastrointestinal cancer types**	**References**
Receptor for Reg3A	gp130	An IL-6 signal transducer	• STAT3 activation in response to Reg3A was almost completely suppressed by pretreating the pancreatic cells with an anti-gp130 neutralizing antibody.	Pancreatic cancer	(38)
	EGFR	Cell proliferation	• The colocalization of Reg3A and EGFR was found in the cytoplasm and membrane of SW1990 cells. • Immunoprecipitation experiments showed Reg3A/EGFR complexes. • Pre-treatment with Erlotinib HCL (EGFR inhibitor) resulted in significant decreases in the pro-proliferation activity of exogenous Reg3A.	Pancreatic cancer	(39)
	EXTL3	Cell proliferation and differentiation	• EXTL3 expressed by keratinocytes was required for the promoting function of Reg3A in the skin keratinocyte proliferation.	–	(10)
	Fibronectin 1	Extracellular matrix protein acting as a stimulator for the phosphorylation of Akt	• The results from co-immunoprecipitation experiments using Reg3A antibody in colorectal cancer LOVO and RKO cells indicated that the endogenous fibronectin 1 and Reg3A were immunoprecipitated by Reg3A antibody, but not by the control IgG.	Colorectal cancer	(32)
			• Reg3A bound strongly to fibronectin 1 in rat hepatocytes.	–	(42)
Downstream targets of Reg3A	JAK2-STAT3	Cell proliferation and death	• Exogenous Reg3A notably enhanced the STAT3 activation by phosphorylation in pancreatic cancer MiaPaCa2 and Panc1 cells.	Pancreatic cancer	(38)
			• STAT3 activation in response to Reg3A was almost completely suppressed by pretreating the pancreatic cells with the JAK2 inhibitor AG-490.		
			• AG-490 treatment to block JAK2 inhibited Reg3A up-regulated expression of pJAK2 and pSTAT3 in pancreatic cancer SW1990, AsPC-1 cells and pancreatic epithelial HPDE6C7 cells.	Pancreatic cancer	(23, 39)
			• *in vivo* treatment with p*Reg3g* in mice increased the expression of pSTAT3 in pancreas tissues.	Pancreatic cancer	(4)
			• After Reg3A knockdown in SGC-7901 cells, the phosphorylations of p-JKA2 and p-STAT3 were markedly decreased, but the total protein expressions of JAK2 and STAT3 were not significantly affected.	Gastric cancer	(27)
	NF-κB		• The induction of NF-κB expression in normal pancreatic epithelial HPDE6C7 cells was obvious after 24 h of exogenous Reg3A exposure.	Pancreatic cancer	(23)
			• *in vivo* treatment with p*Reg3g* in mice increased the expression of NF-κB p65 in pancreas tissues.	Pancreatic cancer	(4)
	SOCS3	A negative feedback inhibitor of cytokine signaling	• The mRNA expression of SOCS3 was significantly lower in Reg3A(–/–) mice.	–	(43)
			• Exogenous Reg3A induced the SOCS3 expression in HPDE6C7, BxPC-3, and PANC-1 cells.	Pancreatic cancer	(23)
			• siRNA-mediated SOCS3 knock-down in normal pancreatic HPDE6C7 cells and plasmid-transfected SOCS3 over-expression in pancreatic cancer cells lead to the obvious promotion and inhibition of Reg3A-induced cell proliferation, respectively.		
			• The *in vivo* transfection of p*Reg3g* in mice resulted in the significant methylation of SOCS3, suggesting SOCS3 methylation might be an important element in the molecular mechanism of Reg3g-driven carcinogenesis.	Pancreatic cancer	(4)
	PI3K/Akt	Proliferation, differentiation apoptosis, invasion	• Using the specific PI3K inhibitors wortmannin and Ly294002 in keratinocytes, PI3K and Akt were identified as the EXTL3-activated downstream effectors in the Reg3A-related signaling pathway	–	(14)
			• The phosphorylation of Akt was significantly suppressed by Reg3A overexpression, whereas, was obviously increased in Reg3A-silenced gastric cancer HGC-27 cells.	Gastric cancer	(40)
			• The inhibitory effect of Reg3A siRNA on the phosphorylation of Akt in LOVO and RKO cells.	Colorectal cancer	(32)
	GSK3β	Proliferation, differentiation apoptosis, invasion	• The phosphorylation of GSK3β was significantly suppressed by Reg3A overexpression, whereas, was obviously increased in Reg3A-silenced gastric cancer HGC-27 cells.	Gastric cancer	(40)
			• CHIR-98014 as the inhibitor of GSK3β abrogated the effect of silenced Reg3A on the cell invasion, proliferation and apoptosis of HGC-27 cells.		
	Erk1/2	Proliferation, metastasis, apoptosis	• The inhibitory effect of Reg3A siRNA on the phosphorylation of Erk1/2 in LOVO and RKO cells.	Colorectal cancer	(32)
	Snail, MMP-2, MMP-9, E-cadherin, RhoC, and MTA1	Invasion and migration-related genes	• Western blot analysis showed that the expression levels of Snail, MMP-2, and MMP-9 were significantly decreased, while those of E-cadherin, RhoC, and MTA1 were significantly enhanced in SGC-7901 cells with Reg3A knockdown.	Gastric cancer	(27)
Upstream molecules of Reg3A	JAK2-STAT3	Cell proliferation and death	• The treatment of AG490 to block JAK2 phosphorylation or siRNA to silence STAT3 lead to a significant decrease in the expression of Reg3A.	Pancreatic cancer	(39)
	Wnt/β-catenin	Cell-cell adhesion	• Reg3A mRNA expression was induced by activation of β-catenin in the Huh7 hepatoma cell line, and this induced expression was abolished by siRNA interference directed against β-catenin.	Hepatocellular carcinoma	(18)

*Akt, Protein kinase B; EGFR, Epidermal growth factor receptor; Erk1/2, Extracellular signal-regulated kinases 1 and 2; EXTL3, Exostosin-like 3; gp130, Glycoprotein 130; GSK3β, Glycogen synthase kinase 3-β; JAK2, Janus kinase 2; MMP, Matrix metalloproteinase; MTA1, Metastasis-associated gene 1; NF-κB, Nuclear factor-κB; PI3K, Phosphatidylinositol 3 kinases; Reg3A, Regenerating islet-derived protein 3A; RhoC, Rho GTPase C; SOCS3, Suppressor of cytokine signaling protein 3; STAT3, Signal transducer and activator of transcription 3; Wnt, Wingless and INT-1*.

## Future Directions

So far, the considerable evidence from patients (17–33) has seemed to be sufficient to support the possible application of Reg3A as a biomarker for gastrointestinal cancer. However, more research is required to determine whether Reg3A as a new biomarker provide an advantage on existing tumor markers. Moreover, based on the specific expression of Reg3A in the digestive system, expression alterations of this molecule in gastrointestinal cancer have attracted attentions from many researchers including us. But few studied (12–14) have reported the existence of Reg3A in extra-intestinal areas such as skin and keratinocytes. Therefore, the possible roles of Reg3A in other types of cancer need to be investigated.

As we reviewed above, the cancer-promoting effects of Reg3A, which were mainly manifested as cell proliferation promotion, cell apoptosis inhibition, the regulation of cancer cell migration and invasion, as well as inflammation-linked pancreatic carcinogenesis, have been established. If this conclusion is confirmed by further work, pharmacological intervention and molecular intervention (using siRNA or small molecules) to suppress the over-expression of Reg3A might be effective for the prevention and treatment of gastrointestinal cancer.

Furthermore, despite some Reg3A-related signal molecules have been identified, further researches are needed to gain a greater insight into the relationship among these molecules. In addition, the previous studies have shown JAK2-STAT3 (39) and Wnt/ β-catenin (18) were responsible for the over-expression of Reg3A in gastrointestinal cancer. However, the exact and comprehensive mechanistic explanation for this over-expression is not yet understood.

## Conclusions

In summary, the overexpression of Reg3A, a protein mainly expressed in the digestive system, has been demonstrated in many kinds of gastrointestinal cancer, including hepatocellular carcinoma, pancreatic cancer, gastric cancer, and colorectal cancer. Up to date, a large amount of evidence have shown that Reg3A mediates diverse functional effects under cancer conditions, including cell proliferation promotion, cell apoptosis inhibition, the regulation of cancer cell migration and invasion. In particular, based on the significant up-regulation of Reg3A during pancreatic inflammation as well as its tumorigenic potential, Reg3A has been believed to play a key role in inflammation-linked pancreatic carcinogenesis. Therefore, Reg3A could be used as a tumor biomarker for gastrointestinal malignancy and as a promising target for prevention and treatment.

## Author Contributions

All authors listed have made a substantial, direct and intellectual contribution to the work, and approved it for publication.

### Conflict of Interest

The authors declare that the research was conducted in the absence of any commercial or financial relationships that could be construed as a potential conflict of interest.
